# Clear Cell Carcinoma of the Endometrium Causing Paraneoplastic Retinopathy: Case Report and Review of the Literature

**DOI:** 10.1155/2011/631929

**Published:** 2011-07-28

**Authors:** Paulina Cybulska, Eduardo V. Navajas, Filiberto Altomare, Marcus Q. Bernardini

**Affiliations:** ^1^Department of Obstetrics and Gynecology, University of Toronto, Toronto, ON, Canada M5G 0A3; ^2^Department of Ophthalmology and Vision Sciences, Princess Margaret Hospital, University of Toronto, Toronto, ON, Canada M5G 2M9; ^3^Division of Gynecologic Oncology, Department of Obstetrics and Gynecology, Princess Margaret Hospital, University of Toronto, Toronto, ON, Canada M5G 2M9

## Abstract

We reviewed the literature for cases in which gynecologic malignancies caused paraneoplastic retinopathy and ultimately led to blindness. Twenty-eight cases were derived from the literature, and one unique case is described from our institution. Of these 28 cases, 14 patients were diagnosed with endometrial cancer, 7 with ovarian cancer, 5 with cervical cancer, 1 fallopian tube cancer and 1 with concomitant endometrial and ovarian cancers. The average age of patients at the time of diagnosis was 64 years (range, 35–89 years). Typically, ocular manifestations antedate symptoms of the underlying carcinoma by 3–12 months. Information regarding the interval from visual symptoms to time of death is limited, but ranges from several months to several years. Our report is the first to document a clear cell carcinoma of the endometrium causing paraneoplastic retinopathy and is the first to review all gynecologic malignancies associated with visual paraneoplastic syndromes.

## 1. Introduction

Paraneoplastic syndromes are characterized by remote effects of a cancer that occur at sites distant from the primary tumor or its metastases. Visual paraneoplastic syndromes are rare and are thought to be mediated by an autoimmune process [1]. Corticosteroids and plasmapheresis are the only treatment options previously described. We review the literature of gynecologic cancers causing visual paraneoplastic syndromes and report the first case of clear cell carcinoma of the endometrium causing paraneoplastic retinopathy.

## 2. Case Report

A 62-year-old woman presented with a 1-year history of postmenopausal bleeding. Preoperative MRI revealed less than 50% myometrial invasion, and she was treated surgically at an affiliated hospital with a total abdominal hysterectomy and left salpingo-oophorectomy. She had previously undergone a right salpingo-oophorectomy. The final pathology revealed a stage 3 clear cell carcinoma with disease penetration to the level of the serosa. Postoperative imaging revealed pelvic and para-aortic lymphadenopathy, and, after discussion at our multidisciplinary rounds, the decision was made to take her back to the operating room for a systematic lymphadenectomy with the hopes of “debulking” and tailoring adjuvant radiation treatment. 

She underwent a robotic assisted pelvic and para-aortic lymphadenectomy from which all thirty lymph nodes removed were found to be negative. After her procedure, due to her high-risk features, she received adjuvant chemotherapy with carboplatin and paclitaxel. The intention was that she receives 6 cycles of chemotherapy followed by pelvic and para-aortic radiation. Unfortunately, she displayed ongoing fatigue and had a drop in hemoglobin averaging 20 gm/L with each treatment resulting in red blood cell transfusion on two occasions. Carboplatin and paclitaxel were both reduced throughout the course of her treatment. Prior to her 4th cycle, she began to complain about “dimmed vision”. CT scans of her chest abdomen and pelvis were repeated at that time. Her previously identified lymphadenopathy had improved, and, due to her overall state, we decided to hold any further chemotherapy. In order to investigate the visual changes, a referral was made to a retinal specialist.

Ophthalmologic examination revealed best corrected visual acuity of 20/150 in both eyes. Anterior segment examination was unremarkable. Fundoscopy demonstrated multiple confluent oval hypopigmented patches at the level of the retinal pigment epithelium (RPE) and a few pigmented choroidal lesions in the posterior pole in both eyes. Fluorescein angiography revealed areas of transmission hyperfluorescence, and optical coherence tomography showed bilateral serous retinal detachment and areas of RPE loss interspersed with zones of thickened RPE. A diagnosis of cancer-associated nummular loss of the pigment epithelium was made.

The patient was treated systemically with five sessions of plasmapheresis and intravenous immunoglobulin, and locally with bilateral subtenons triamcinolone acetonide injection and an intravitreal triamcinolone acetonide injection in the left eye. Despite treatment, eight months after presentation, vision deteriorated to light perception only in both eyes. She was found to have further loss of RPE, enlargement of one pigmented choroidal lesion, and persistent retinal detachments. Twelve months after presentation cataracts developed as well as iris-pigmented lesions. 

Five months after the completion of initial chemotherapy and after investigations and treatment for visual loss, the patient underwent another staging CT scan. This scan showed an increase in the size of the patient's retroperitoneal lymph nodes, and a fine needle aspiration did confirm recurrent metastatic disease 13 months after the initial surgery was performed. After consultation with medical oncology, it was deemed that she would not tolerate conventional chemotherapy nor was she a candidate for clinical trials. She was started on Megace 80 mg twice daily. Unfortunately, she continued to show signs of progressive disease and 21 months after her initial operation was discharged to hospice care.

## 3. Discussion

Bilateral diffuse uveal melanocytic proliferation (BDUMP) is a rare paraneoplastic syndrome, characterized by bilateral vision loss in patients with a systemic carcinoma [24]. Our patient was diagnosed with a rare variant of BDUMP, named cancer-associated nummular loss of the retinal pigment epithelium. It is the only reported case of clear cell adenocarcinoma of the uterus associated with a paraneoplastic syndrome. BDUMP is characterized by uveal tract thickening and degeneration and atrophy of the retinal pigment epithelium [21] and usually does not respond to corticosteroid treatments [24] as was the case with our patient. 

There were 28 previously reported cases of gynecologic cancers associated with paraneoplastic retinopathy (Table 1). Of these, 14 patients were diagnosed with endometrial cancer, 7 with ovarian cancer, 5 with cervical cancer, 1 with fallopian tube cancer, and 1 with concomitant endometrial and ovarian cancers. The average age of patients at the time of diagnosis was 64 years (range, 35–89 years). Information regarding the interval from visual symptoms to time of death is limited but can range from several months to several years. At the time of publication, our patient is alive in hospice care, 16 months after her initial visual complaint, and 21 months after her cancer diagnosis was made.

Paraneoplastic retinopathy has been related to other cancer types, including lung and various GI malignancies (bowel, gallbladder, pancreas). Typically, ocular manifestations antedate symptoms of the underlying carcinoma by 3–12 months [9, 21]. However, our patient and the majority of gynecologic cases (15/28) have visual symptoms following the diagnosis of cancer. Classically, patients commonly complain of flickering lights, reduced visual acuity, color impairment and photosensitivity [28]. Our patient complained of “darkness” and visual “dimming.” 

The onset of visual symptoms during treatment for a gynecologic malignancy should alert the oncologist to a possible cancer-associated retinopathy. A referral to the ophthalmologist is warranted.

## Figures and Tables

**Table 1:** Reported gynecologic cancers associated with paraneoplastic retinopathy.

Reference	Age at diagnosis	Origin of cancer	Interval between diagnosis of primary tumor and onset of visual symptoms (months)	Interval from visual symptoms to death (months)
[2]	62	Endometrioid sarcoma	+5	7
[3]	77	Poorly differentiated adenocarcinoma of the ovary	−23	23
[4]	60	Papillary cystadenocarcinoma of ovary	+7	NA
[5]	50	Uterine cancer	+36	No information
[6]	89	Poorly differentiated carcinoma of ovary	−15	15
[7]	61	Undifferentiated neoplasm of the cervix uteri	−7	20
[8]	64	Undifferentiated endometrial adenocarcinoma	Initial diagnosis: +8 recurrence: −3	3
[9]	68	Carcinoma of the cervix	−3	12
	65	Serous cystadenocarcinoma of ovary	+24	Well after 78 months
[10]	63	Clear cell carcinoma of ovary	−5	8
[11]	72	Uterine small cell carcinoma	−2	6
[12]	65	Malignant mixed mullerian tumor (homologous) of the endometrium	−1	NA
[13]	77	Anaplastic uterine carcinoma	+10	NA
[14]	60	Endometrial carcinoma	Initial diagnosis: −3 recurrence: −11	27
[15]	67	Small-cell endometrial carcinoma	+12	4
[16]	35	Carcinoid tumour of cervix	Article in german	
[17]	60	Mixed-type small cell carcinoma of endometrium	Initial diagnosis: −4 recurrence: −18	30
[18]	55	Clear cell carcinoma of ovary	+27	4
[19]	75	Poorly differentiated adenocarcinoma of the ovary	−11	Not stated; patient died after 6 courses of chemotherapy
[1]	77	Adenocarcinoma cervix	−0.5 –1	NA
[20]	35	Serous ovarian cancer and endometrial carcinoma	Initial diagnosis: +24 recurrence: +1	6
[21]	70	Uterine cancer	+12	54
[22]	58	Small cell undifferentiated carcinoma with minor components of endometroid and mucinous grade 1/3 of the endometrium	−1	2
[23]	57	Uterine small cell carcinoma	−1.5	NA
[24]	66	Endometrial carcinoma	+4	NA
[25]	67	Endometrial carcinoma	Concomitant	NA
[26]	68	Adenocarcinoma of cervix	−48	NA
[27]	70	Neuroendocrine carcinoma of fallopian tube	−22	NA
Current	62	Clear cell carcinoma of the uterus	+8	NA

The interval between diagnosis of the inciting tumor and the diagnosis of retinopathy is listed as positive numbers if the inciting tumor was diagnosed before the diagnosis of retinopathy and as negative numbers if the inciting tumor was diagnosed after the diagnosis of retinopathy.

NA: patient alive at the time of publication.
